# Exploring neuropsychological alterations in VEXAS syndrome

**DOI:** 10.1007/s10238-026-02283-4

**Published:** 2026-08-18

**Authors:** Sara Bindoli, Chiara Ceolin, Maria Devita, Camilla Marrone, Davide Spinetti, Marina De Rui, Daniela Mapelli, Andrea Doria, Roberta Ramonda, Giuseppe Sergi, Paolo Sfriso

**Affiliations:** 1 Department of Medicine (DIMED), University of Padova, Padova, Italy; 2 Rheumatology Unit, Padova University Hospital, Padova, Italy; 3 Geriatrics Division, Padova University Hospital, Padova, Italy; 4 Department of Neurobiology, Care Sciences and Society, Aging Research Center, Karolinska Institutet and Stockholm University, Stockholm, Sweden; 5 Department of Psychology (DPG), University of Padova, Padova, Italy; 6 Department of Surgery, Oncology, Gastroenterology (DiSCOG), University of Padova, Padova, Italy

**Keywords:** VEXAS, Neuropsychology, Neuroinflammation, Monogenic diseases, Inflammation

## Abstract

VEXAS syndrome (Vacuoles, E1 enzyme, X-linked, Autoinflammatory, Somatic) is an adult-onset “hemato-autoinflammatory” disease provoked by somatic mutations in the *UBA1* gene within hematopoietic progenitor cells. While dermatologic, rheumatologic and hematologic manifestations are well-documented, its neurological and cognitive implications remain poorly characterized. Given the chronic inflammatory burden of VEXAS, this study aimed to explore cognitive functioning in affected individuals. In this retrospective pilot study, we performed a neuropsychological assessment in patients with genetically confirmed VEXAS, VEXAS-like individuals (VEXAS-compatible symptoms without *UBA1* variations), patients with mild cognitive impairment (MCI), and healthy controls (HC). Participants underwent computerized attention testing (Cognitrone) and multidomain cognitive evaluation (ENB-2), with analyses adjusted for age and education. Thirty-four participants were included: 7 patients with genetically confirmed VEXAS syndrome, 5 VEXAS-like patients, 11 individuals with mild cognitive impairment (MCI), and 11 healthy controls (HC). Mean age ranged from 63.3 ± 10.3 years in the VEXAS-like group to 75.7 ± 6.9 years in the VEXAS group. After adjustment for age and education, VEXAS-like patients showed cognitive performances broadly comparable to those of healthy controls. Patients with VEXAS demonstrated lower performance on selected measures of attentional control and response monitoring, whereas individuals with MCI showed broader cognitive impairment. Measures related to memory interference and complex attention displayed limited differences across groups. This pilot study represents the first standardized neuropsychological evaluation of patients with VEXAS syndrome. The findings suggest the presence of subtle differences in selected cognitive domains, warranting confirmation in larger longitudinal studies integrating inflammatory biomarkers and neuroimaging measures.

**Supplementary Information:**

The online version contains supplementary material available at https://doi.org/10.1007/s10238-026-02283-4.

## Introduction

VEXAS syndrome (Vacuoles, E1 enzyme, X-linked, Autoinflammatory, Somatic) is an adult-onset hematologic-autoinflammatory disorder, caused by somatic loss-of-function mutations in the *UBA1* gene (Xp11.3), within hematopoietic progenitor cells [1]. Recent population-based estimates suggest a prevalence of approximately 1 in 4,000 men and 1 in 26,000 women over the age of 50 years, indicating that VEXAS may be more common than initially recognized, despite remaining a rare disorder [2]. Three causal variations that change the start codon of the cytoplasmic isoform in exon 3 were found in the first description (M41T, M41L, and M41V) [1], but splice site variants have also been recognized [3] to disrupt of the function of E1 ubiquitin-activating enzyme, resulting in a profound alteration of the ubiquitination process, ultimately manifesting as a heterogeneous clinical spectrum [1]. Patients typically present with systemic inflammatory features such as fever, neutrophilic dermatosis, vasculitic-like skin lesions, nasal and/or auricular chondritis, inflammatory arthritis, pulmonary infiltrates and serositis [4], along with hematologic abnormalities of the myeloid lineage, most notably macrocytic anemia and myelodysplastic syndrome (MDS) [5].

To date, several studies have detailed the most prevalent clinical features observed during the course of VEXAS syndrome. Cutaneous involvement is one of the most common features, reported in up to 89% of patients, and it is predominantly characterized by neutrophilic dermatosis and arcuate lesions [6]. Chondritis is observed in approximately 60% of patients [5]. Musculoskeletal manifestations, including inflammatory arthritis and myositis, have been reported with variable frequency due to their clinical heterogeneity [5, 7]. Pulmonary involvement occurs in 50% to 72% of cases, presenting with diverse radiologic findings such as pulmonary nodules, septal thickening, ground-glass opacities, and pleuritis [8]. Among haematological features, macrocytic anaemia is present in nearly all affected individuals, while MDS represents the most frequently associated hematologic malignancy. Thrombotic complications are also commonly reported, including those affecting the central nervous system (CNS), such as cerebral venous sinus thrombosis, as recently reported [9].

Neurological and neuropsychological involvement in VEXAS syndrome remains poorly described in the current literature. Given the high systemic inflammatory burden characteristic of this condition, a comprehensive evaluation of its potential impact on cognitive functioning is warranted. The concept of neuroinflammation is well established in disorders with a primary neuroinflammatory aetiology, such as multiple sclerosis (MS), as well as in conditions with a well-defined autoimmune basis, including neuromyelitis optica (NMO) and autoimmune encephalitis [10]. In contrast, other neuroinflammatory conditions appear less driven by autoreactive lymphocytes and more associated with dysregulated innate immune responses, as exemplified by Behçet’s disease [11]. Within the autoinflammatory spectrum, a wide range of systemic autoinflammatory diseases (SAID) may present with a CNS involvement, spanning classical periodic fever syndromes [e.g. Familial Mediterranean Fever (FMF), Tumor necrosis factor Receptor-Associated Periodic Syndrome (TRAPS), Mevalonate Kinase Deficiency (MKD), and Cryopyrin-Associated Periodic Syndrome (CAPS)], mainly mediated by IL-1β, to type I interferonopathies and NF-κB–related disorders. Neurological involvement, however, in IL-1β–mediated periodic fever syndromes is generally limited. In FMF indeed, headache and encephalitis have been described, but remain rare [12] likewise in TRAPS and MKD [10]. By contrast, CAPS, particularly the CINCA/NOMID phenotype and Muckle–Wells syndrome, are more frequently associated with neurological manifestations, including headache (30–80%), sensorineural hearing loss (40–70%), and papilledema (approximately 30% [13, , 15]. The higher prevalence of these features compared with other periodic fever IL-1β-mediated, supports a potential role for NLRP3 inflammasome activation in microglial dysfunction, thereby contributing to neuroinflammation and increasing susceptibility to CNS injury, including infection and cerebrovascular events [16].

A distinct group of SAID is represented by type I interferonopathies, characterized by excessive IFNα/β signaling [17]. First described by Aicardi and Goutières, these disorders are linked to pathogenic variants in at least nine recognized genes, to date [18]. Aicardi–Goutières syndrome (AGS) is defined by early-onset severe neurological dysfunction, intracranial calcifications, leukoencephalopathy, cerebral atrophy, CSF pleocytosis, and cutaneous involvement. Although type I interferon overactivation consistently induces neuropathology, marked phenotypic variability suggests additional modifying factors, especially in the late-onset forms in adults [10].

Similarly, polyarteritis nodosa (PAN), a sporadic disease more frequently observed in adults, and adenosine deaminase 2 deficiency (DADA2), a predominantly pediatric disorder caused by biallelic variants in the *ADA2* gene, but increasingly recognized in adult-onset cases, both present as medium-vessel necrotizing vasculitides with overlapping clinical features, including hematologic and skin involvement. In addition, both conditions may involve the central nervous system, particularly through ischemic strokes, and peripheral nervous system involvement (e.g. mononeuritis multiplex, polyneuritis and peripheral neuropathy). Therefore, within the spectrum of type I interferon–mediated inflammatory syndromes, these entities, in particular PAN, should be systematically considered in the differential diagnosis of VEXAS, given their clinical overlap and potential for diagnostic confusion [19] .

Among hematological autoinflammatory conditions, secondary hemophagocytic lymphohistiocytosis (sHLH) is particularly relevant, as it may occur as main complications in SAIDs such as Still’s disease [20], although sHLH occurring after VEXAS syndrome has been recently described [21]. sHLH is characterized by an intense cytokine storm, implying a significant possible neuroinflammatory burden and justifying early treatment with high-dose dexamethasone and IL-1 blockade (e.g., anakinra), both of which can cross the blood–brain barrier (BBB) and mitigate a potential CNS inflammation [22] .

NF-κB signaling instead, represents a central regulator of inflammatory burden across multiple conditions, including Behçet’s disease, haploinsufficiency of A20, and other SAIDs [10]. VEXAS syndrome is also considered an NF-κB–driven disease. In the CNS, NF-κB is constitutively expressed and regulates key functions in neurons, glia, and cerebral vessels [23]. NF-κB activation has also been implicated in perioperative neurocognitive disorders (PND), a condition characterized by alterations in behavior, emotion, and cognition triggered by anesthesia and surgery, where it amplifies systemic inflammation, disrupts BBB, and promotes oxidative stress, ultimately leading to neuronal injury and cognitive dysfunction [24]. Immune and glial cells further potentiate this process through NF-κB–dependent pathways. The example of PND provides a rationale for why several secondary triggers, including thrombotic events, stroke, and infections, might further exacerbate neuroinflammation thereby contributing to a possible neurocognitive impairment in patients with VEXAS syndrome.

Overall, given the high degree of systemic inflammation that characterizes autoinflammatory diseases, these conditions may be associated with a highly heterogeneous neuroinflammatory burden, reflecting differences in their underlying molecular and immunopathogenic pathways. Conditions driven by type I interferon or NF-κB signaling likely involve more prominent CNS involvement compared with IL-1β–mediated disorders. In this context, VEXAS syndrome lies within an NF-κB-dominant inflammatory spectrum. Additionally, the hematological involvement, together with inflammaging and clonal hematopoiesis, may contribute to create a permissive microenvironment for persistent cytokine production, thereby amplifying and sustaining central neuroinflammatory responses.

For these reasons, the present study aimed to address this gap by comprehensively characterizing a cohort of patients with genetically confirmed VEXAS syndrome (harboring canonical *UBA1* mutations) alongside a group of VEXAS-like patients, defined as individuals presenting with clinical features consistent with VEXAS syndrome but lacking canonical *UBA1* mutations. To further investigate the underlying pathogenic mechanisms in these patients, all underwent targeted myeloid next-generation sequencing to identify possible somatic mutations associated with clonal hematopoiesis and systemic inflammatory states. These groups were compared with a control cohort consisting of individuals without systemic inflammation but diagnosed with mild cognitive impairment (MCI), and healthy controls (HC), to investigate how chronic inflammation may influence neuropsychological performance.

## Patients and methods

Given the rarity of VEXAS syndrome, this exploratory pilot study retrospectively enrolled thirty-four subjects. Seven male patients with genetically confirmed VEXAS syndrome (UBA1-mutated), five VEXAS-like individuals without detectable UBA1 mutations, eleven patients with MCI, and HC were included. Patients with VEXAS syndrome were consecutively recruited from the Autoinflammatory Clinic of Padova University Hospital, Italy. VEXAS-like individuals presented with clinical manifestations suggestive of VEXAS syndrome, but tested negative for *UBA1* mutations. Patients with MCI were recruited from the Geriatric Unit of the same institution and met standard diagnostic criteria for MCI, including objective cognitive impairment in at least one domain, preserved activities of daily living, and absence of dementia. Healthy controls were community-dwelling volunteers, matched for age and sex, without a history of autoimmune, inflammatory, neurological, or psychiatric disorders, and were recruited through routine health screenings or community outreach programs. Individuals with established dementia, major psychiatric disorders, severe sensory deficits interfering with neuropsychological testing, or acute medical conditions at the time of assessment were excluded. All participants gave their written informed consent to participate in the study, which was conducted in compliance with the principles of the Declaration of Helsinki. The protocol follows the guidelines of the Ethics Committee of Padova University Hospital (protocol code CET 6362/AO/25).

### Genetic and clinical assessment

Whole blood samples were collected from patients for exome sequencing following informed consent. Notably, *UBA1* mutations in HC and MCI were not screened. Libraries were prepared using the *Agilent SureSelect All Exon* V6 kit and sequenced with *Illumina HiSeq2500* or *NextSeq2000* platforms. All variants were classified based on American College of Medical Genetics and Genomics (ACMG) guidelines. Clinical data of VEXAS and VEXAS-like subjects were recorded in electronic databases during patient visits to the Outpatient Clinic for Autoinflammatory diseases.

### Neuropsychological assessment

All neuropsychological assessments were conducted individually in a dedicated, quiet, well-lit, and temperature-controlled room, free from external distractions. Testing was administered face-to-face by the same trained neuropsychologist following standardized procedures and the administration instructions provided in the Cognitrone and ENB-2 manuals. Whenever possible, assessments were scheduled preferably in the morning, to reduce variability related to circadian fluctuations in cognitive performance. Before testing, participants were asked about current fatigue, pain, sleep quality, acute discomfort, regular medication use, and the timing of their last medication intake; deviations from the usual medication schedule were recorded. Visual and hearing abilities were checked before test administration, and participants were allowed to use their habitual corrective aids, such as glasses or hearing devices. Short breaks were offered when signs of fatigue emerged, particularly given the age and clinical characteristics of the sample. A detailed description of tests administered follows below:

- The Cognitrone (COG-S3) is a computer-based test developed by Schuhfried GmbH as part of the Vienna Test System (WTS) [25]. It assesses concentration and attention by measuring the ability to compare figures and make decisions quickly and accurately under time pressure. Participants must decide whether a comparison figure matches one of several reference figures and respond accordingly. The test is sensitive to cognitive fatigue, processing speed, and attentional lapses. Variable Descriptions: WTS_RG_S3 – Number of Correct Responses (Correct Acceptances). This is the number of correct responses the participant gave when the comparison figure matched one of the reference figures. It reflects processing accuracy and speed under pressure. A lower score may indicate attentional lapses or slowed cognitive processing. WTS_NR_S3 – Number of Non-Responses. This measures how often the participant failed to respond within the given time window. High values suggest slowness in decision-making, potential uncertainty, or cognitive overload. WTS_FA – Number of False Alarms (Incorrect Acceptances). This reflects the number of times a participant incorrectly identified a non-matching figure as a match. It is a measure of impulsivity, inhibitory control deficits, or impaired stimulus discrimination. WTS_RI_S3 – Number of Incomplete Reactions. These are responses that were initiated (e.g., a key was pressed) but not completed or executed properly. High scores may reflect motor response inconsistencies, attention instability, or premature responses that are not fully processed.
- The “Esame Neuropsicologico Breve” 2 (ENB, Short Neuropsychological Examination-2) [26] is a standardized Italian neuropsychological screening battery developed to assess global cognitive functioning across a range of domains. It is particularly suited for use in both clinical and research contexts, offering a time-efficient yet comprehensive evaluation of cognitive performance in adult and older-adult populations. The battery includes 14 subtests that probe various cognitive domains such as attention, working memory, verbal and non-verbal memory, executive functioning, language comprehension and production, visuospatial abilities, praxis, and orientation. Performance on the ENB-2 provides a multidimensional cognitive profile, with specific subtests assessing core functions like digit span, verbal recall (immediate and delayed), interference sensitivity, trail making, verbal fluency, and visuoconstructive skills. Additionally, tasks such as the clock drawing test and cognitive estimation offer insights into higher-order planning and abstract reasoning. Importantly, each subtest has normative references adjusted for age and education, allowing for precise interpretation of individual and group-level cognitive status. The ENB-2 has been shown to be sensitive to mild cognitive impairment and early changes in cognitive function, making it a valuable tool for detecting subtle neuropsychological alterations in diverse clinical populations.

### Statistical analyses

Data distributions were assessed using the Shapiro–Wilk test to evaluate normality. Depending on the distribution, results are presented as either mean ± standard deviation or median with interquartile range. Group comparisons were conducted using Analysis of Covariance (ANCOVA) to assess differences across diagnostic groups (HC, VEXAS-like, VEXAS, and MCI), with age and years of education included as covariates to control for potential confounding factors.

Separate ANCOVA models were performed for each cognitive variable. Raw p-values are reported, and effect sizes (partial eta squared) are provided to quantify the magnitude of group differences. Given the exploratory nature of this pilot study, no formal correction for multiple comparisons was applied.

For variables showing *p* < 0.10, visual inspection of score distributions was carried out using boxplots. The threshold for statistical significance was set at *p* < 0.05. All analyses were conducted using IBM SPSS Statistics version 29 and Python (v3.10) for advanced plotting and data visualization.

## Results

### Sample characteristics

Clinical features and genotypes of VEXAS and VEXAS-like patients included in the study are summarized in Table 1. When comparing HC, individuals with MCI, and patients with VEXAS or VEXAS-like syndromes, no significant differences were observed among the groups in either age or years of education. Mean age was 72.5 ± 9.0 years in healthy controls, 73.7 ± 10.1 in MCI, 63.3 ± 10.3 in VEXAS-like patients, and 75.7 ± 6.9 in VEXAS patients (*p* = 0.19). Similarly, years of education did not differ significantly across groups, with means of 10.82 ± 3.22 in healthy controls, 11.00 ± 3.58 in MCI, 11.00 ± 2.40 in VEXAS-like patients, and 10.17 ± 4.92 in VEXAS patients (*p* = 0.97).

**Table 1:** General overview of the clinical manifestations and genotypes of VEXAS and VEXAS-like patients included in the study

	UBA1 genotype	VAF (%)	Constitutional symptoms	Domain							
				Hematologic *	Thrombosis (DVT/SVT)	Skin #	Musculoskeletal §	Ocular ¥	Chondritis	Lung €	PNS/CNS=
VEXAS	c.121 A > C p. Met41Leu	90%	✔	✔	✔	✔	✔		✔	✔	
	c.118–2 A > G	74.6%	✔	✔	✔	✔	✔		✔	✔	✔
	c.121 A > G p. Met41Val	62.5%	✔	✔	✔	✔	✔	✔			
	c.121 A > G p. Met41Val	32.5%	✔		✔	✔		✔			
	c.122 C > T p. Met41Thr	60%	✔	✔	✔	✔	✔	✔	✔	✔	✔
	c.121 A > G p. Met41Val	20.3%	✔	✔	✔			✔		✔	
	c.118-1G > C	51.3%	✔	✔	✔		✔		✔	✔	
VEXAS-like											
	Negative		✔	✔		✔	✔				
	Negative		✔	✔			✔	✔	✔		
	Negative		✔	✔			✔				
	Negative		✔			✔	✔	✔	✔		✔
	Negative		✔	✔		✔	✔	✔	✔		

Hematologic features including: myelodysplastic syndrome (MDS), monoclonal gammopathy uncertain significance (MGUS), macrocytic anaemia; # Skin features including neutrophilic dermatosis, Sweet syndrome like, cutaneous vasculitis (e.g. livedo, purpuric rash, erythema nodosum); § Musculoskeletal features including: arthritis, arthralgia, myalgia and/or myositis manifestations; PNS: peripheral nervous system; CNS: central nervous system; VAF: variant allele frequency

### Cognitive performance across diagnostic groups

Group comparisons on cognitive performance are presented in Table 2. After adjustment for age and education, significant overall group differences were observed for selected indices derived from the Cognitrone task, particularly measures related to response accuracy and response execution. In contrast, measures of memory interference and complex attention showed limited differences across groups.

**Table 2:** Cognitive performance across diagnostic groups (Healthy, MCI, VEXAS, VEXAS-like)

Variable	Healthy (*n* = 11)	VEXAS-like (*n* = 4)	VEXAS (*n* = 7)	MCI (*n* = 11)	ANCOVA *p*-value	Partial Eta Squared
WTS RG S3	16.00 ± 0.00	15.75 ± 0.50	14.80 ± 1.10	10.50 ± 7.80	***0.048***	0.422
WTS NR S3	0.00 ± 0.00	0.00 ± 0.00	0.80 ± 0.84	1.50 ± 2.12	*0.073*	0.383
WTS FA	0.00 ± 0.00	0.25 ± 0.50	0.20 ± 0.45	1.00 ± 1.41	*0.084*	0.368
WTS RI S3	0.00 ± 0.00	0.50 ± 1.00	0.20 ± 0.50	5.50 ± 7.80	***0.039***	0.44
ENB2 Interferenza10sec	8.00 (6.50–9.00)	9.00 (7.75–9.00)	8.00 (4.00;9.00)	7.00 (5.00–9.00)	0.858	0.03
ENB2 Interferenza30sec	8.00 (7.00–9.00)	6.50 (3.75;7.75)	6.00 (3.00;8.00)	8.00 (4.50–9.00)	0.218	0.172
ENB2 TMTB	97.00 (83.00–125.00)	101.00 (67.75;177.00)	135.00 (118.00;192.00)	99.00 (64.00;120.00)	0.627	0.113
ENB2 Orologio	9.50 (8.50–10.00)	10.00 (9.00–10.00)	9.50 (7.50–10.00)	9.50 (8.50–10.00)	0.709	0.055
WTS TR S3	535.00 ± 106.82	558.50 ± 45.21	572.40 ± 66.65	640.50 ± 173.24	0.311	0.219
WTS TM S3	243.33 ± 76.62	180.75 ± 33.19	251.80 ± 40.07	334.50 ± 204.35	0.537	0.139

Values are presented as mean ± standard deviation or median (IQR), depending on the distribution of each variable, assessed using the Shapiro–Wilk test. P-values were obtained from ANCOVA models, including age and education as covariates. Partial Eta Squared indicates the proportion of variance explained by the group effect. For WTS RG S3, higher scores indicate better performance. For WTS NR S3, WTS FA, and WTS RI S3, higher scores indicate a greater number of errors or incomplete responses and therefore poorer performance.

**Fig. 1:**
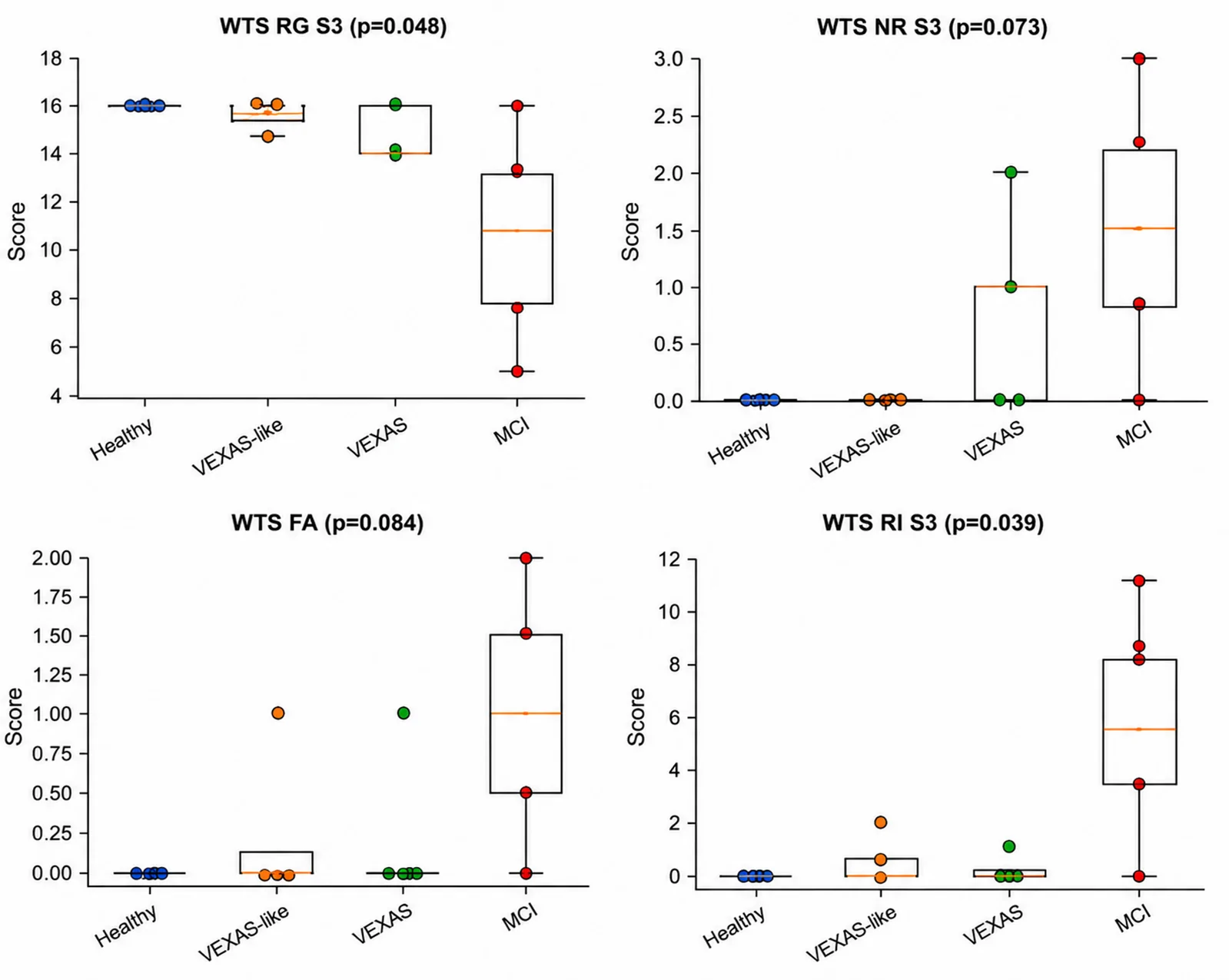
Distribution of scores on the main Cognitrone (Vienna Test System) variables across the four study groups. *Notes*: p-values reflect the overall group effect. No multiple comparison correction was applied

Figure 1 illustrates the distribution of raw scores for the principal WTS variables across the four study groups.

**Fig. 2:**
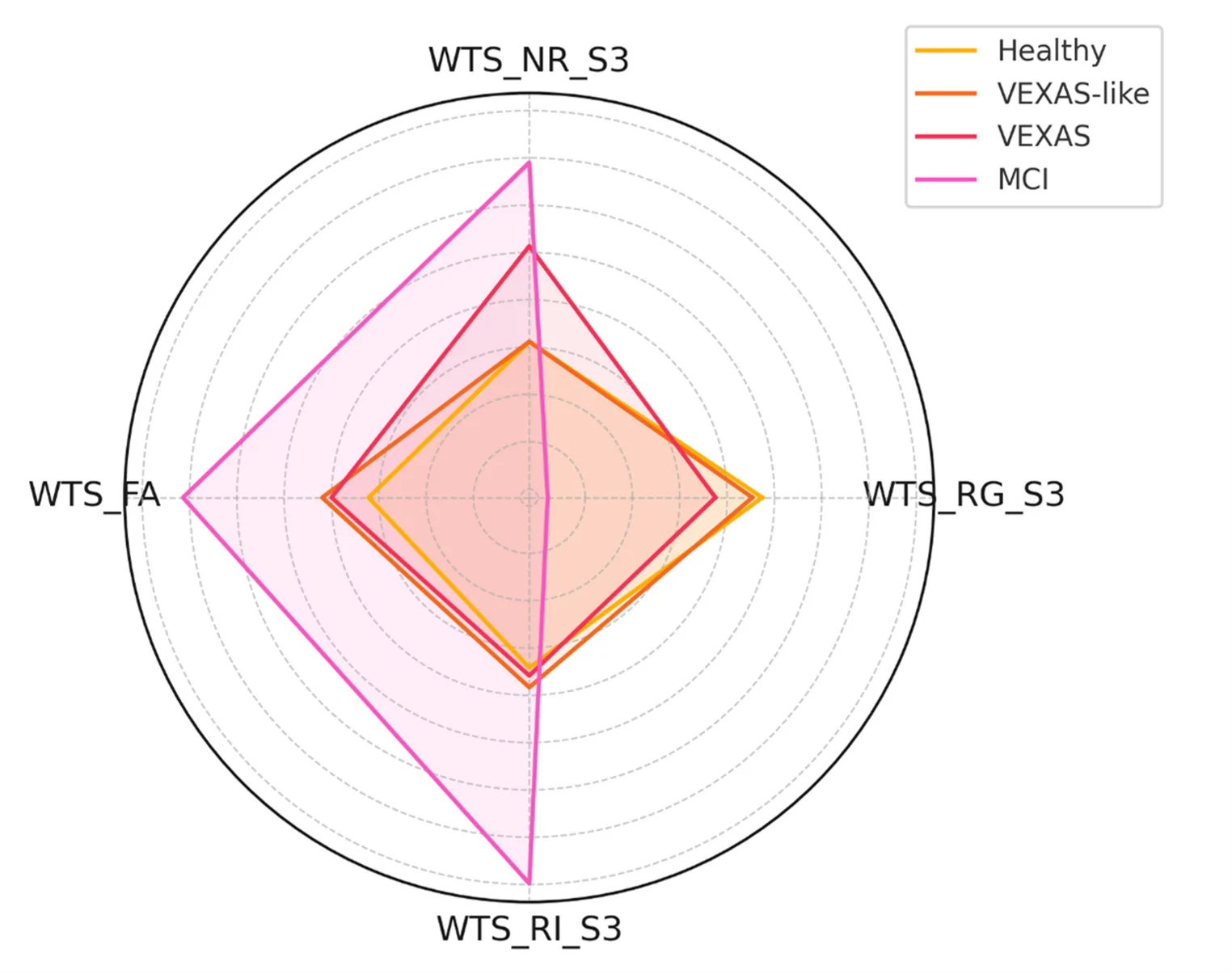
Standardized Cognitive Profiles Across Groups Radar plot illustrating the standardized cognitive performance (z-scores) across four key WTS variables (WTS_RG_S3, WTS_NR_S3, WTS_FA, WTS_RI_S3) for MCI, VEXAS, VEXAS-like, and healthy control groups. Higher values indicate better performance or fewer errors. Higher values indicate better performance (i.e., higher scores or fewer errors). Each line represents the mean standardized score of the corresponding group. Across all four executive domains assessed with WTS tasks, VEXAS patients consistently exhibited intermediate performance, falling between the MCI group and the other two groups (Healthy and VEXAS-like), which performed similarly to each other

**Fig. 3:**
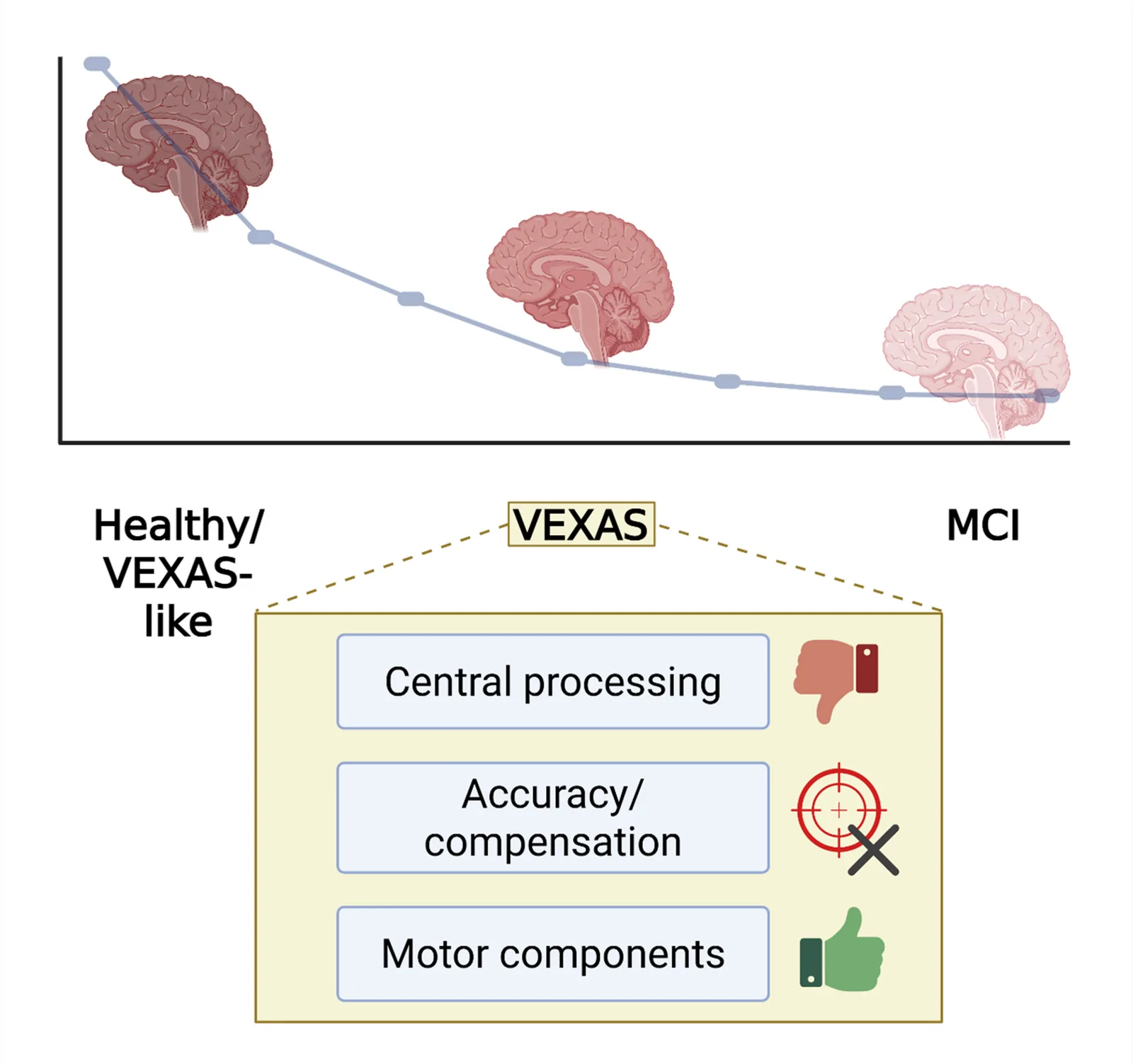
Conceptual representation of the cognitive continuum across clinical groups. A visual model illustrating the hypothetical position of VEXAS on a continuum between healthy aging and mild cognitive impairment (MCI). Brain schematics reflect progressive neurofunctional deterioration. The inset highlights the VEXAS cognitive profile: impaired central processing, reduced compensatory accuracy mechanisms, and preserved motor components

## Discussion

Although neurological manifestations were not part of the initial description of VEXAS syndrome, emerging evidence suggests they occur in a small, yet likely underrecognized, subset of patients. These manifestations are notably diverse, affecting both the CNS and peripheral nervous system (PNS). A detailed analysis by Bert-Marcaz et al., using data from the French VEXAS Registry involving 291 patients, reported neurological involvement in 6% of cases [27]. Among those affected, PNS involvement predominated, in 70% of cases, and included both axonal and demyelinating neuropathies. The clinical spectrum is broad, with presentations such as multiple mononeuropathies and non-length-dependent neuropathies, exhibiting chronic, acute, or subacute onset. Importantly, cranial nerve palsy was a prominent finding, affecting nearly one-third of patients with PNS involvement [27].

While CNS involvement in VEXAS syndrome appears to be less common than PNS manifestations, it is similarly varied in presentation and may be linked to increased mortality. Reported CNS cases have all exhibited acute or subacute onset, with confusion and cognitive impairment as the predominant symptoms. Neuroimaging has revealed findings such as extensive white matter abnormalities and lacunar ischemic lesions, pointing toward a potential association between VEXAS and cerebral small vessel disease. Notably, a recent case of CNS vasculitis has been documented [28]. Since white matter lesions are frequently seen in older adults and are associated with heightened risks of stroke, cognitive decline, and depression, their presence in individuals with VEXAS may carry important clinical consequences [27]. Other rare neurological manifestations may include optic neuritis, aseptic meningitis, inflammatory pseudotumor, and rhombencephalitis [29].

The emerging concept of neuroinflammation in rheumatological disorders, including conditions like fibromyalgia and several SAIDs IFN- and NFkB-mediated [10], suggests that cognitive dysfunction in these patients may arise from neuroinflammatory processes driven by diverse pro-inflammatory pathways, such as microglial activation, elevated cytokine levels (e.g., IL-1β, IL-6, TNF-α), and blood–brain barrier disruption, ultimately leading to impaired synaptic plasticity and neuronal signalling [30], although elevated systemic cytokines does not necessarily establish direct neuroimmune activation of both CNS and PNS. However, considering the multifaceted nature of VEXAS, and particularly its hematological involvement, it is reasonable to view the relationship among neurological impairment, aging, inflammation, and clonal hematopoiesis (CHIP) as complex and bidirectional. The causal interplay between “inflammaging” and CHIP may therefore be central to understanding the pathogenesis of myelodysplastic syndromes (MDS), which is often associated with VEXAS. Indeed, lifelong exposure to a wide range of antigens and immune stressors progressively contributes to the development of the pro-inflammatory phenotype characteristic of inflammaging. In addition, several factors, including diet, physical activity, and other environmental exposures, act as modulators capable of perturbing this process [10]. Recently, a model of VEXAS-associated inflammaging has been proposed, whereby loss-of-function mutations in the *UBA1* gene impair the initial step of ubiquitin activation and its subsequent conjugation to target proteins via E2 and E3 enzymes, leading to the accumulation of misfolded and aberrant proteins. This, in turn, triggers the unfolded protein response (UPR) through activation of the PERK, IRE1α, and ATF6 pathways, ultimately resulting in enhanced type I interferon production. This mechanism differs from the classical model of pre-clonal inflammaging, in which the aged bone marrow microenvironment becomes progressively pro-inflammatory, thereby facilitating the emergence of clonal hematopoiesis and the progression to myelodysplastic syndromes [31]. The proposed framework may provide a potential explanation for the high inflammatory burden observed in VEXAS and raises the possibility of potential CNS involvement, although this hypothesis remains untested. These mechanisms may also parallel those implicated in Alzheimer’s disease (AD), thereby potentially exacerbating or accelerating age-related cognitive decline [32].

Indeed, the dysfunction of the ubiquitin-proteasome system (UPS) is a process implicated in Alzheimer’s disease. The accumulation of misfolded proteins, which in turn triggers the release of pro-inflammatory cytokines, contributing to neurodegeneration. Consequently, circulating pro-inflammatory cytokines may cross the BBB, where they can activate glial cells and drive neuroinflammatory responses [32, 33]. It should be mentioned that in VEXAS syndrome, vascular complications could theoretically contribute to cerebral hypoperfusion and cognitive dysfunction in some patients. Given that macrocytic anemia is a nearly universal feature in VEXAS, it is also important to consider that chronic anemia may result in reduced oxygen delivery, leading to persistent fatigue, decreased exercise capacity, and impaired cognitive function, including reduced attention. Additionally, there is growing evidence to suggest that specific CHIP mutations such as *TET2*,* DNMT3A* [34], *JAK2* or *TP53* [35], observed both in VEXAS and VEXAS-like patients variably correlate with cardiovascular disease progression [31]. This pathophysiological interplay could represent one of several hypothetical mechanisms potentially contributing to the observed findings, although this possibility was not directly investigated in the present study. Growing evidences indicate that bone marrow (BM) failures and myeloid malignancy predisposition syndromes are characterized by a wide phenotypic spectrum and that these diseases must be considered in the differential diagnosis of children and adults with unexplained hematopoiesis defects [36].

Delving further into the inflammatory profile, type I interferons, markedly elevated in VEXAS are especially harmful to the neuronal environment, where they play a key role in microglial activation and sustaining neuroinflammation. The pattern of results across tasks may suggest subtle differences in information processing speed and attentional-executive performance in patients with VEXAS syndrome, particularly under time pressure. Tasks such as WTS_RG_S3 and WTS_RI_S3, which tap into processing accuracy, attentional stability, and premature responding, showed lower performance in patients with VEXAS, although these findings should be interpreted cautiously given the exploratory nature of the study and the limited sample size. Even in measures where differences were only borderline significant (WTS_RS_FA and WTS_NR_S3), a trend toward progressively lower performance across groups was observed—healthy controls and VEXAS-like individuals scoring highest, VEXAS patients showing intermediate performance, and MCI patients the lowest. This pattern may indicate subtle differences in cognitive processing efficiency that are not typical of healthy aging yet do not reach the severity observed in neurodegenerative conditions. These findings may suggest subtle differences in information processing and response monitoring, although the underlying neurobiological mechanisms cannot be inferred from the present data. Such tasks are known to engage attentional and executive processes that have been linked to distributed cortical and subcortical networks. Emerging evidence suggests that elevated levels of pro-inflammatory cytokines such as IL-6 and TNF-α—known to cross the BBB—can interfere with neural signaling, impair synaptic plasticity, and disrupt white matter integrity [37, 38]. These mechanisms are believed to contribute to functional alterations in neural circuits, particularly those involving the frontal lobes and basal ganglia, which are essential for attentional control, response selection, and psychomotor coordination [39]. Findings from neuroimaging studies in other chronic inflammatory conditions (e.g., lupus, rheumatoid arthritis, systemic vasculitis) support this view, revealing prefrontal hypoactivation and altered connectivity in networks such as the default mode and salience networks [40]. Although direct neuroimaging data in VEXAS are lacking, the present findings are compatible with hypotheses linking chronic systemic inflammation to alterations in cognitive efficiency, a possibility that warrants further investigation. The relative sparing of performance on traditional executive tasks—such as the Trail Making Test or standard interference measures—may reflect preserved compensatory mechanisms that suffice under conditions of low complexity, but become inadequate when temporal precision and executive load increase [41]. This interpretation is further supported by the observed variability and reduced performance stability across tasks, suggesting a pattern of non-degenerative, yet functionally meaningful, cognitive vulnerability. Such deficits, while not always severe, can have downstream effects on higher-order functions like working memory, reasoning, and adaptive behavior [42, 43]. Under time constraints, the reduced capacity to efficiently process and integrate information may lead patients to adopt compensatory strategies—such as being overly cautious or responding prematurely—depending on task demands. Importantly, these measures may prove useful for detecting subtle cognitive differences in VEXAS and deserve further evaluation in larger studies. However, given the small sample size and exploratory nature of the study, no firm conclusions can be drawn regarding the nature or clinical relevance of these findings [44, 45]. Such an intermediate cognitive profile aligns with the concept of a continuum between normative aging and early neurocognitive decline. Crucially, these findings underscore an important methodological consideration. In our cohort, dynamic computerized tasks appeared to capture group differences that were less evident on traditional neuropsychological measures. Whether these approaches are more sensitive to inflammation-related cognitive changes requires confirmation in larger studies. In contrast, dynamic paradigms assessing reaction time, decision accuracy, and error monitoring in real time—such as those employed in this study—may provide a more precise and ecologically valid assessment of cognitive functioning in inflammatory syndromes like VEXAS. Importantly, the present study did not include neuroimaging, inflammatory biomarkers, or direct measures of BBB integrity. Therefore, any biological mechanisms proposed to explain the observed cognitive findings should be considered speculative and hypothesis-generating rather than demonstrated by the current data. Additionally, all the patients (both VEXAS and VEXAS like) received glucocorticoids (GCs). GCs are known to exert significant effects on the CNS and may contribute to cognitive dysfunction, mood disturbances, and broader neuropsychiatric alterations, thus representing a potential confounding factor in the interpretation of our findings [46]. However, accurately quantifying the extent to which steroid therapy contributes to the neuropsychological profile observed in our patients remains challenging. Notably, given that these are relatively recently diagnosed cases, the cumulative corticosteroid burden in terms of both dose and duration is not yet substantial, although it should be acknowledged that some patients had been exposed to steroid therapy prior to receiving a definitive diagnosis. Nevertheless, this represents an important limitation of the present study. Furthermore, a careful review of the clinical history revealed that none of the patients reported a clear temporal association between the initiation of GCs therapy and the onset of psychological or behavioral changes. For this reason, while we fully acknowledge the potential confounding role of GCs, particularly their well-documented capacity to impair cognitive function and alter neuropsychological performance, we consider it unlikely that steroid exposure represents the primary driver of the neuropsychological profile observed in this cohort at the current stage of disease and treatment.

### Limitations and strengths

Several limitations should be acknowledged in this study. First, the very small sample size, inherent to the rarity of VEXAS syndrome, substantially limits statistical power and restricts the ability to draw robust conclusions. This limitation is particularly relevant when comparing four groups and adjusting for covariates such as age and education. Although ANCOVA was retained to allow adjustment for these clinically relevant covariates, the very small and unbalanced group sizes limit the robustness of parametric assumptions and increase uncertainty around the estimates. Consequently, the present findings should be considered exploratory and hypothesis-generating and require confirmation in larger multicenter cohorts. Additionally, due to the limited number of participants, it was not feasible to perform stratified analyses based on different genotypes. This is particularly relevant given that certain variants, such as *UBA1* p.Met41Val, have been associated with a more severe disease course and poorer prognosis [5]. The MCI group likely represented a clinically heterogeneous population, and detailed information regarding MCI subtypes and associated comorbidities was not systematically available because of the retrospective design. This heterogeneity may have contributed to the greater variability observed in cognitive performance within this group. Moreover, current evidence remains insufficient to conclusively demonstrate impairment in specific executive functions among VEXAS and VEXAS-like patients. In our study, no statistically significant differences were observed in any of the evaluated cognitive domains or in global cognitive performance between VEXAS and VEXAS-like groups, although VEXAS subjects tended to show poorer performance. Interestingly, VEXAS-like patients, who present inflammatory features overlapping with the classical one observed in VEXAS, might probably display an involvement of other alternative inflammatory pathways. It is important to note that VEXAS-like individuals in this study did not undergo comprehensive next-generation sequencing (NGS) for other SAIDs or whole-exome sequencing (WES), but they underwent only an NGS myeloid panel, leaving open the possibility that other genetic mutations may contribute to their inflammatory phenotype. Interestingly, one VEXAS-like patient harbored an *IDH1* variant, which has recently been described as a novel driver of a VEXAS-like phenotype [47]. Another important limitation concerns the lack of systematic assessment of potential neuropsychological confounders. Mood disorders, fatigue, chronic pain, sleep disturbances, and anxiety are common in chronic inflammatory conditions and may influence cognitive performance, particularly processing speed and executive functioning. As these variables were not systematically collected, their potential contribution to the observed findings cannot be excluded. Future studies incorporating validated measures of mood, fatigue, and sleep quality are warranted to better characterize the determinants of cognitive dysfunction in VEXAS syndrome. Another important limitation is the lack of inflammatory biomarker measurements obtained contemporaneously with the neuropsychological assessment. Consequently, the relationship between systemic inflammatory activity and cognitive performance could not be directly examined. Furthermore, although variant allele frequency (VAF) may represent a biologically relevant marker of clonal burden, the limited number of patients with available VAF data precluded meaningful analyses of the relationship between allele burden and neuropsychological performance. Exploratory correlation analyses did not identify significant associations; however, these findings should be interpreted with caution given the very limited statistical power. Future studies including larger cohorts will be needed to clarify whether clonal expansion contributes to the severity or pattern of cognitive impairment in VEXAS syndrome.

Despite these limitations, the study also presents several strengths. To our knowledge, this is the first investigation to specifically assess cognitive function in patients with VEXAS syndrome, framing the question within the broader context of a potential cognitive continuum between healthy aging and mild cognitive impairment. This innovative perspective guided the choice of a multidimensional neuropsychological assessment, encompassing both classical cognitive tests and dynamic paradigms capable of capturing subtle changes in processing speed, executive control, and cognitive variability. The inclusion of a matched healthy control group and a clinical comparison group with MCI adds further robustness to the design, allowing for a more nuanced interpretation of the cognitive profile associated with VEXAS. Furthermore, by integrating insights from neuroimmunology and cognitive neuroscience, the study contributes to an emerging field of inquiry that seeks to understand how chronic systemic inflammation may influence brain function. In this sense, the study addresses a largely unexplored aspect of VEXAS syndrome and provides a foundation for future research with larger cohorts and longitudinal designs that may clarify the trajectory and potential reversibility of cognitive alterations in VEXAS syndrome.

## Conclusions

This pilot study provides preliminary evidence of subtle differences in selected aspects of attentional control and cognitive processing in patients with VEXAS syndrome. These findings should be interpreted with caution and warrant confirmation in larger longitudinal studies integrating inflammatory biomarkers and neuroimaging measures. Future studies may help clarify whether dynamic cognitive assessments provide additional insights into the neurocognitive manifestations of VEXAS and whether cognitive alterations represent a stable trait, a fluctuating state, or a potentially modifiable target for intervention. Additionally, this study may pave the way for the incorporation of routine neuropsychological assessments in clinical practice, with the aim of identifying recurrent cognitive pathways and shared neurocognitive profiles within patient subgroups. Such an approach could ultimately support patient stratification, potentially allowing clustering based on variables such as inflammatory or cardiovascular burden. While these considerations remain speculative, neuropsychological assessment is likely to play a crucial role in characterizing the broader background and heterogeneity of the disease, contributing to a more comprehensive understanding of its clinical phenotype.

## Supplementary Information

Below is the link to the electronic supplementary material.

Supplementary Material 1

## Data Availability

The datasets generated and analyzed during the current study are not publicly available due to privacy reasons but are available from the corresponding author on reason request.
