# Supplementary material for: Exploring neuropsychological alterations in VEXAS syndrome
Source: Clin Exp Med. 2026 Aug 18;26(1):378. doi: 10.1007/s10238-026-02283-4 (PMC13759599; doi:10.1007/s10238-026-02283-4)
Supplement: Supplementary file 1 [file 10238_2026_2283_MOESM1_ESM.docx]

**Supplementary Table S1. Clinical, therapeutic and laboratory features of VEXAS and VEXAS-like patients included in the study**

| VEXAS pz | *UBA1* genotype | VAF (%) | Diagnostic delay (years) | Previous diagnosis | Previous therapies | Current therapy | Transfusion dependency | Hematologic Support  & DVT Treatment | Thrombotic events | Hb/MCV (worst values or during flares) | CRP (mean values over time) | Infections |
| --- | --- | --- | --- | --- | --- | --- | --- | --- | --- | --- | --- | --- |
| 001 | *c.121 A>C p.(M41L)* | 90% | 3 | Vasculitis | GC, MTX, MPDN, Filgotinib | PDN | Yes | EPO; DOAC | Yes | Hb 80 g/L MCV 100.4 fL | 40 mg/l | Sepsis and urosepsis (recurrent episodes) |
| 002 | *c.118-2>G* | 74.6% | 6 | RP | GC, MTX, colchicine,  Filgotinib | GC, Pacritinib | No | DOAC | Yes | Hb 120 g/L  MCV 110 fL | 30 mg/l | Sepsis (recurrent episodes) |
| 003 | *c.121 A>G p.(M41V)* | 62.5% | 1 | PMR | GC, MTX, colchicine | GC, MTX | No | No | No | Hb 132 g/L MCV 98 fL | 5 mg/l | Sepsis; endocarditis |
| 004 | *c.121 A>G p.(M41V)* | 32.5% | Unknown | FUO | Na | Na | No | No | No | Na | Na | No |
| 005 | *c.122 C>T p.(M41T)* | 60% | 7 | RP | GC, MTX, MMF, AZA | GC, Filgotinib | No | DOAC | Yes | Hb 89 g/l  MCV 102 fL | 30 mg/L | Sepsis (recurrent episodes); SARS-CoV-2 severe infection |
| 006 | *c.121A>G p.(M41V)* | 20.3% | 7 | Behçet Disease/Vasculitis | GC | GC, Pacritinib | No | VKA | Yes | Hb 138g/L MCV 105 fL | 20 mg/l | No |
| 007 | *c.118-1G>C* | 51.3% | 3 | CTD  /PMR/  CPPD | GC,colchicine, MTX | (patient deceased) | Yes | DOAC | Yes | Hb 89 g/L  MCV 110 fL | 40 mg/l | Osteomyelitis |
| VEXAS-like pz | **Other non-*UBA1* variants** |  | | | | | | | | | | |
| 008 | *IDH1* | 13% | - | Sweet Syndrome | GC, anakinra, colchicine | GC | No | No | No | Hb 115 g/L  MCV 103 fL | 80 mg/L | No |
| 009 | *Myeloid panel ongoing* | _ | - | FUO | GC | Na | No | No | No | Hb 134 g/L MCV 102 fL | 10 mg/l | No |
| 010 | *SF3B1* | na | - | MDS with fever | GC | Na | No | No | No | Hb 109 g/L, MCV 116 fL | 20 mg/l | No |
| 011 | *Myeloid panel ongoing* | - | - | RP | GC, MTX | GC, MTX | No | No | No | Hb 130 g/L, MCV 106 fL | 5 mg/l | No |
| 012 | *ASXL1*  *EZH2* | 23.4 %  5.2% | - | Sweet Syndrome | GC, dapsone, ruxolitinib, IgIV | 5-Azacytidine | Yes | EPO | Yes | Hb 134g/L, MCV 110 fl | 30 mg/L | Sepsis/Cellulitis |

**Legend:**CPPD: Calcium Pyrophosphate Deposition; CRP: C-Reactive Protein; CTD: Connective Tissue Disease; DOAC; Direct Oral Anti Coagulant; DVT: Deep vein thrombosis; EPO: Erythropoietin; FUO: Fever of unknown origin; GC: Glucocorticoids; Hb: haemoglobin; IgIV: Intravenous Immunoglobulins; MCV: Mean Corpuscular Volume; MDS: Myelodysplastic syndrome; MPDN: Methylprednisolone; MTX: Methotrexate; PDN: Prednisone; PMR: Polymyalgia Rheumatica; RP: Recurrent Polychondritis; VKA: Vitamin K Antagonists

**Supplementary Table S2. Cardio-vascular (CVD) risk factors of VEXAS and VEXAS-like patients included in the study**

| **Patients/CVD risk factors** | **Diabetes** | **Hypertension** | **Dyslipidemia** | **Smoke (current or past)** | **Atrial Fibrillation** | **Coronary Artery Disease Hystory** |
| --- | --- | --- | --- | --- | --- | --- |
| **VEXAS 001** | 0 | 0 | 0 | 0 | 1 | 0 |
| **VEXAS 002** | 0 | 0 | 0 | 0 | 1 | 0 |
| **VEXAS 003** | 0 | 0 | 0 | 0 | 0 | 0 |
| **VEXAS 004** | 0 | 0 | 0 | 0 | 0 | 0 |
| **VEXAS 005** | 0 | 1 | 1 | 1 | 0 | 0 |
| **VEXAS 006** | 1 | 1 | 0 | 0 | 0 | 0 |
| **VEXAS 007** | 0 | 0 | 0 | 0 | 0 | 0 |
| **VEXAS-like 008** | 0 | 0 | 0 | 0 | 0 | 0 |
| **VEXAS-like 009** | 0 | 0 | 0 | 0 | 0 | 0 |
| **VEXAS-like 010** | 0 | 0 | 0 | 0 | 0 | 0 |
| **VEXAS-like 011** | 0 | 0 | 0 | 0 | 0 | 0 |
| **VEXAS-like 012** | 1 | 1 | 0 | 1 | 0 | 0 |

Legend: 1: yes; 0: no.
